# Differences in Brain Morphological Findings between Narcolepsy with and without Cataplexy

**DOI:** 10.1371/journal.pone.0081059

**Published:** 2013-11-28

**Authors:** Masaki Nakamura, Shingo Nishida, Kenichi Hayashida, Yoichiro Ueki, Yves Dauvilliers, Yuichi Inoue

**Affiliations:** 1 Japan Somnology Center, Neuropsychiatric Research Institute, Tokyo, Japan; 2 Tokyo Medical University, Department of Somnology, Tokyo, Japan; 3 National Reference Network for Narcolepsy, Sleep-Disorders Center, Department of Neurology, Hôpital Gui de Chauliac, Inserm U1061, UM1, Montpellier, France; Hospital General Dr. Manuel Gea González, Mexico

## Abstract

**Objective:**

Maps of fractional anisotropy (FA) and apparent diffusion coefficient (ADC) obtained by diffusion tensor imaging (DTI) can detect microscopic axonal changes by estimating the diffusivity of water molecules using magnetic resonance imaging (MRI). We applied an MRI voxel-based statistical approach to FA and ADC maps to evaluate microstructural abnormalities in the brain in narcolepsy and to investigate differences between patients having narcolepsy with and without cataplexy.

**Methods:**

Twelve patients with drug-naive narcolepsy with cataplexy (NA/CA), 12 with drug-naive narcolepsy without cataplexy (NA w/o CA) and 12 age-matched healthy normal controls (NC) were enrolled. FA and ADC maps for these 3 groups were statistically compared by using voxel-based one-way ANOVA. In addition, we investigated the correlation between FA and ADC values and clinical variables in the patient groups.

**Results:**

Compared to the NC group, the NA/CA group showed higher ADC values in the left inferior frontal gyrus and left amygdala, and a lower ADC value in the left postcentral gyrus. The ADC value in the right inferior frontal gyrus and FA value in the right precuneus were higher for NA/CA group than for the NA w/o CA group. However, no significant differences were observed in FA and ADC values between the NA w/o CA and NC groups in any of the areas investigated. In addition, no correlation was found between the clinical variables and ADC and FA values of any brain areas in these patient groups.

**Conclusions:**

Several microstructural changes were noted in the inferior frontal gyrus and amygdala in the NA/CA but not in the NA w/o CA group. These findings suggest that these 2 narcolepsy conditions have different pathological mechanisms: narcolepsy without cataplexy form appears to be a potentially broader condition without any significant brain imaging differences from normal controls.

## Introduction

Narcolepsy is a disabling sleep disorder characterized by excessive daytime sleepiness (EDS) and abnormal rapid eye movement(REM) sleep manifestations [1]. Dysregulation of the hypocretinergic system plays an important role in the pathogenesis of human narcolepsy-cataplexy [2], [3].

Some Previous neuroimaging studies have attempted to clarify the mechanisms of the brain dysfunction in narcolepsy. For example, in a study by Asenbaum et al., single photon emission computed tomography (SPECT) showed increased blood flow in the right hemisphere and decreased blood flow in the thalamus during REM sleep in patients with narcolepsy [4]. In another study that used positron emission tomography (PET), cerebral perfusion was found to be reduced in the bilateral anterior hypothalami, prefrontal cortices, cingulated gyri, and many other midbrain structures [5]. Furthermore, cerebral glucose Hypometabolism in the hypothalamic-thalamic-orbitofrontal pathways in narcolepsy was reported by Joo et al [6]. These studies have indicated that dysfunction of the midbrain and prefrontal cortices might be associated with narcolepsy pathology, however, the results were still conflicting.

With regard to brain morphological studies on narcolepsy-cataplexy, Desseilles et al. reported volumetric changes mainly in hypothalamic gray matter and/or the frontal cortex [7]. However, Overeem et al. reported no differences in hypothalamic volume between patients with hypocretin-deficient narcolepsy and controls [8]. Thus, brain morphometric changes in narcolepsy are still under debate.

In the second edition of the International Classification of Sleep Disorders (ICSD-2), narcolepsy was divided into 2 separate entities: i.e., narcolepsy with and without cataplexy. Narcolepsy without cataplexy differs from the former condition not only in terms of the absence of cataplexy but also in clinical (less severe EDS), biological (low CSF-hypocretin-1 levels in 10–20% of cases) and neurophysiological (lesser increase of REM sleep propensity) characteristics [3], [9]–[11]. A histopathological study indicated that hypocretin fiber density in the anterior hypothalamus was decreased in narcolepsy with cataplexy, but it was normal in narcolepsy without cataplexy [12]. This finding may suggest that the differences in clinical characteristics between narcolepsy with and without cataplexy are related to the difference in hypocretin fiber density in the anterior hypothalamus. However, all previous neuroimaging narcolepsy studies were conducted exclusively in patients with narcolepsy with cataplexy. To the best of our knowledge, brain morphological and microstructural differences have never been studied in patients having narcolepsy with and without cataplexy.

Diffusion tensor imaging (DTI) can identify the microstructural changes in neurons, as indicated by the values obtained for fractional anisotropy (FA) and apparent diffusion coefficient (ADC) [13]. FA values represent tract directionality and integrity of neuronal fibers, being sensitive to the number, coherence, and degree of myelination of neural fibers [13]. ADC values reflect microscopic water molecule diffusivity, and a high ADC value is thought to represent an increase in extracellular space and indicates microstructural changes in neurons [13]. In the current study, we applied a voxel-based statistical approach to FA and ADC maps of patients having narcolepsy with and without cataplexy to evaluate brain microstructural differences.

## Methods

This study was approved by the ethics committees of the Neuropsychiatric Research Institute. All participant provided written informed consent before entering this study.

Twelve Japanese drug-naive patients having narcolepsy with cataplexy (NA/CA), 12 drug-naive patients having narcolepsy without cataplexy (NA w/o CA) and 12 non-obese healthy controls without EDS (NC) matched for age and sex were enrolled from April 2008 to December 2009 (Table 1). NA/CA and NA w/o CA were diagnosed according to the criteria outlined in the ICSD-2 [14]. To ensure accurate diagnoses, cataplexy symptoms were confirmed through detailed clinical interviews by 2 or more board-certified physicians who are specialized in sleep medicine. All patients underwent multiple sleep latency test (MSLT) following nocturnal polysomnography (PSG). Mean latencies of both sleep and REM sleep onset were analyzed in 4 or 5 nap trials on MSLT, according to a standard method [15]. All patients were asked to record their sleep time on sleep logs during a 2-week period before PSGs to ensure that they had no sleep insufficiency and that they all had slept for more than 6 hours on all the PSG nights. None of the patients had sleep apnea or periodic limb movements during sleep (apnea-hypopnea index<5/h, periodic limb movements index<15/h,) or any medical disorders that could cause narcolepsy-like sleepiness. All subjects had normal findings on physical and neurological examinations, and they had no comorbid psychiatric disorders, including depression and REM sleep behavior disorder.

**Table 1:** Clinical and neurophysiological data of patients having narcolepsy with and without cataplexy.

	Narcolepsy with cataplexy	Narcolepsy without cataplexy	Normal Control
Sample size	12	12	12
Male: female	9∶3	6∶6	6∶6
Age (years)	29.4±4.9	26.0±5.2	29.8±2.2
Age range (years)	(22–40)	(20–35)	(26–34)
Body mass index (BMI)	24.0±2.4*	21.3±1.8	
JESS score	18.5±3.1	17.9±5.1	
MSLT parameters			
Sleep onset latency (minutes)	1.1±0.6*	2.6±1.7	
Latency of Sleep onset REM (minutes)	3.7±3.4	5.7±3.4	
Frequency of SOREM period (%)	90.1±15.6	81.3±18.8	
Onset age of EDS (years)	18.2±7.4	16.0±3.4	
Duration of narcolepsy (years)	11.3±4.6	10.0±7.3	
Sleep paralysis	10/12	8/12	
Hypnagogic/hypnopompic hallucinations	12/12	8/12	
Apnea hypopnea index (per hour)	3.1±2.4	2.1±3.3	
Periodic legs movement index (per hour)	4.1±7.2	0.2±0.6	

Continuous values are expressed as mean±SD.

*p<0.05(t-test).

JESS: Epworth Sleepiness Scale, Japanese version.

MSLT: multiple sleep latency test.

### MRI acquisition

MRI was performed using a 1.5T (GE Medical Systems) scanner with a conventional head coil. The T1-weighted images were obtained by a spin-echo sequence using the following parameters; repetition time (TR), 1,930 msec; echo time (TE), 13 msec: inversion time, 900 ms; flip angle 90°;, and matrix size, 512×512. DTI was performed by a fat suppressed spin-echo type single-shot echo-planer with a TR of 11,000 ms, a TE of 81 and slice thickness of 3.5 mm, without an interslice gap. Motion probing gradients (MPGs) for DTI were applied in 17 noncollinear directions (b = 1000 s/mm^2^) after the acquisition of b = 0 s/mm^2^ (b0) images. The transverse slices were aligned to the anterior-posterior commissure(AC-PC line) line. All subjects underwent brain MRI examinations during the daytime, in an awake condition, before receiving treatments.

### FA and ADC maps preprocessing and statistics

The DTI data were transferred to an off-line windows PC with a Intel Core2 Extreme Quad CPU (2.27GHz) and 8.0 GB of memory for post-processing. FA and ADC maps were calculated using the dTV-II (Image Computing and Analysis Laboratory, Department of Radiology, University of Tokyo Hospital, http://www.ut-radiology.umin.jp/people/masutani/dTV.htm) [16] and VOLUME-ONE software. MRI T2-weighted template was used to spatially normalize these FA and ADC maps. The normalized FA and ADC maps were smoothed with FWHM (full-width-half-maximum) of 12 mm to increase the signal/noise ratio. These normalizing and soothing methods were implemented according to the spatial normalization and smoothing procedure included in SPM8 (Wellcome Department of Imaging Neuroscience, Institute of Neurology, London, UK) running in MATLAB 7.11 R2010b (The MathWorks; Natick, MA, USA).

Each normalized and smoothed FA and ADC maps were compared by using voxel-based between-subjects one-way ANOVA in SPM8 with global normalization by whole brain volume as a covariate among NA/CA, NA w/o CA and NC groups. An absolute threshold mask of 0.2 was used to avoid possible edge effects around the border between the gray and white matter. The significance level was set at p <0.001 (uncorrected) for peak level on ANOVA. After conducting the ANOVA, in order to investigate significant main effects, multiple voxel-wise comparisons between NA/CA and NA w/o CA, between NA/CA and NC, and between NA w/o CA and NC were performed with post-hoc tests in cluster-level using family-wise error corrected p value (P_FWE-corr_) <0.05 to protect against the occurrence of overall Type I Error. Gaussian random fields theory and Bonferroni procedure were also used to control the FWE rate by assuming that the data follow certain specified patterns of spatial variance and that the statistical distributions mimic a smoothly varying random field.

Correlation analyses were performed for the NA/CA group by using voxel-based multiple regression analysis in SPM8 with a significance level of p <0.05 in cluster level between significant regions detected with FA/ADC maps. Correlation analyses were also performed with respect to age, gender, duration of narcolepsy, age at onset, frequency of cataplexy, sleepiness score as assessed by the Japanese version of the Epworth sleepiness scale(JESS) [17], and both sleep onset latency and the number of sleep onset REM periods (SOREMPs) on MSLT.

## Results

### Clinical and demographic data

Mean age and gender distribution were similar between the NA/CA, NA w/o CA and NC groups. Age at onset of EDS, duration of NA, mean JESS score, sleep onset REM (SOREM) latency and frequency of SOREMP period on MSLT (percentage of naps with SOREMP per total number of naps) did not differ between NA/CA and NA w/o CA groups (Table 1). Body mass index (BMI) was higher and sleep onset latency on MSLT was shorter in the NA/CA group than in the NA w/o CA group. Ten out of 12 patients having NA/CA reported to have sleep paralysis (83.3%), and all 12 patients had episodes of hypnagogic/hypnopompic hallucinations. Among the 12 patients having NA w/o CA, sleep paralysis and hypnagogic/hypnopompic hallucinations were reported by 8 (66.7%) and 7 (58.4%) patients, respectively. All patients were drug-naive at the time of PSG and MRI examinations.

### Comparison of brain images among narcolepsy with cataplexy, narcolepsy without cataplexy and normal controls

**Figure 1:**
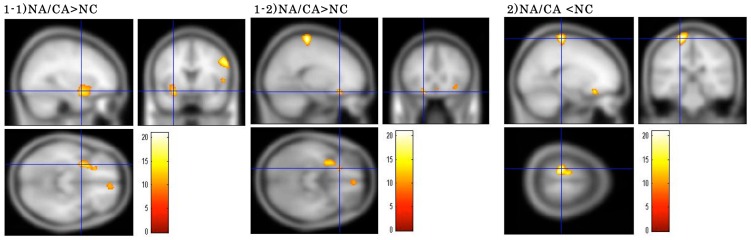
Clusters showing significant main effects of group on ADC value between patients having narcolepsy with cataplexy (NA/CA) and normal control (NC). 1-1) ADC values were higher in the left parahippocampal gyrus (Brodmann area 34) and amygdala, and 1-2) in the left inferior frontal gyrus (Brodmann area 47/11) in NA/CA than in NC, while these values were lower in 2) the left postcentral gyrus (Brodmann area 3) in the former group. pone.0081059.Results.tifare significant at FWE-corrected p<0.05. Color scale is for F statistic.

**Figure 2:**
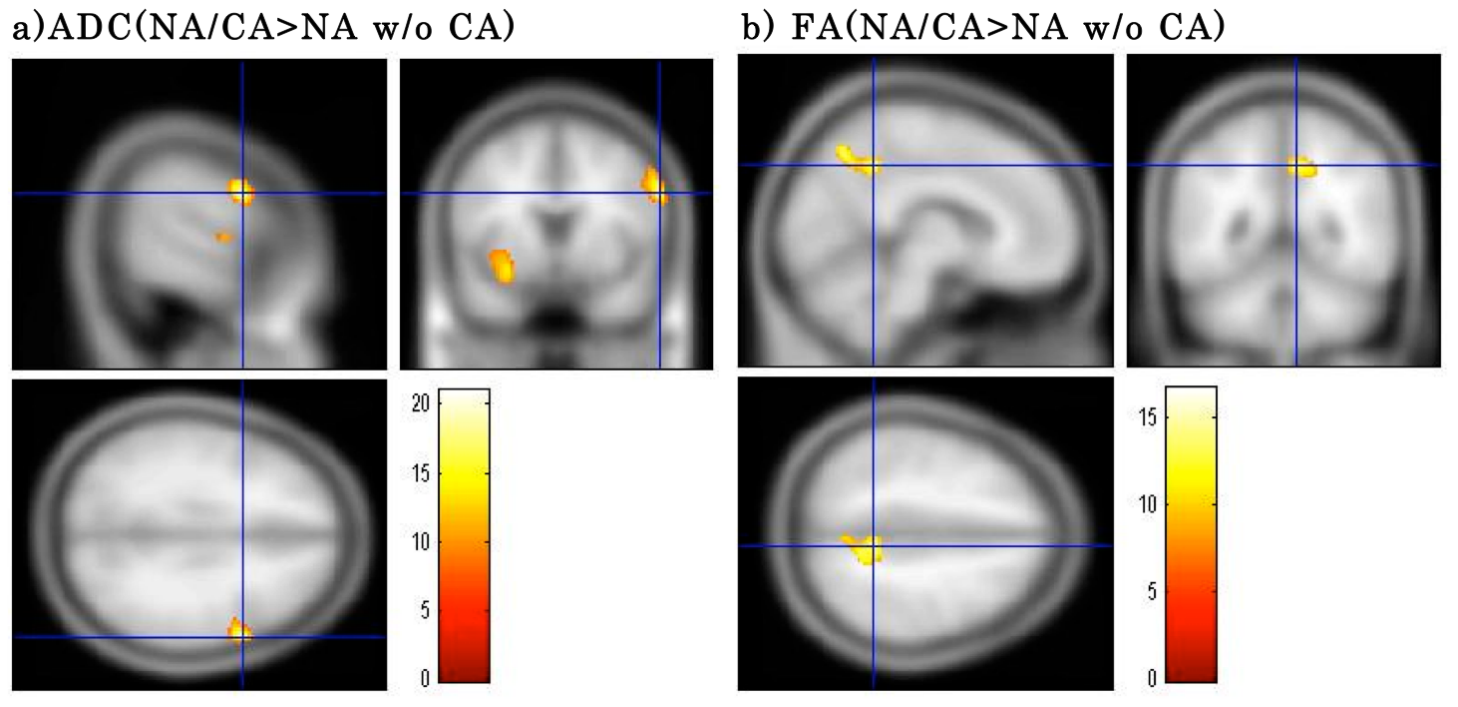
Clusters showing significant main effects of group on ADC and FA values between the narcolepsy with cataplexy (NA/CA) and narcolepsy without cataplexy (NA w/o CA) groups. a) ADC value was higher in the right inferior frontal gyrus (Brodmann area 9) for the NA/CA group than for the NA w/o CA group. b) In the NA/CA group, the FA value in the right parietal lobe (precuneus) was higher than that in the NA w/o CAgroup. Results are significant at FWE-corrected p<0.05. Color scale is for F statistic.

SPM-one-way ANOVA for the NA/CA, NA w/o CA and NC groups revealed significant differences in ADC values for the right inferior frontal gyrus [F(2,32) = 17.76, uncorrected p<0.001], left inferior frontal gyrus [F(2,32) = 14.17, uncorrected p<0.001], left parahippocampal gyrus and amygdala [F(2,32) = 13.90, uncorrected p<0.001], and left postcentral gyrus [F(2,32) = 21.00, uncorrected p<0.001]. The results of post-hoc tests with multiple voxel-wise comparisons indicated that the ADC value for the right inferior frontal gyrus was higher in the NA/CA than in the NA w/o CA group (P_FWE-corr_ = 0.028), and ADC values for the left inferior frontal gyrus, left parahippocampal gyrus and amygdala (P_FWE-corr_ = 0.000) were heigher in the NA/CA group compared to the NC group (Table 2, Figure 1, 2). In contrast, the ADC values for the left postcentral gyrus were lower in the NA/CA than in the NC group (P_FWE-corr_ = 0.015). FA values for the right parietal lobe (precuneus) significantly differed between the 2 NA groups [F(2,32) = 13.87, uncorrected p<0.001], with higher value in the NA/CA than in the NA w/o CA group on post-hoc test (P_FWE-corr_ = 0.008) (Table 2, Figure 2). No significant differences were found in the ADC, or FA values for any brain areas between the NA w/o CA and NC groups.

**Table 2:** Significant differences in the ADC and FA values for the NA/CA, NA w/o CA, and NC groups.

			Brodmann area	Talairach coordinate		Cluster-level	
Contrast	Image	Anatomical Region		x, y, z	F	P_FWE-corr_	P_uncorr_
NACA>NC	ADC	Left inferior frontal gyrus	47,11	−20, 26, −18	14.17	0.000	0.000
		Left parahippocampal gyrus/amygdala	34	−30, 4, −14	13.90	0.000	0.000
NACA<NC	ADC	Left postcentral gyrus	3	−20, −30, 72	21.00	0.015	0.002
NACA>NA w/o CA	ADC	Right inferior frontal gyrus	9	60, 8, 30	17.76	0.028	0.004
	FA	Right parietal lobe (precuneus)	7	8, −48,44	13.87	0.008	0.001

Height threshold uncorrected p<0.001 in peak level on ANOVA.

Group main effects in cluster-level by multiple voxel-wise comparisons using P_FWE-corr_: family-wise error, corrected p.

NA/CA: narcolepsy with cataplexy, NA w/o CA: narcolepsy without cataplexy, NC: normal controls.

ADC: apparent diffusion coefficient, FA: fractional anisotropy.

Finally, no significant correlations were found between variables such as age, gender, duration of narcolepsy, age at onset, frequency of cataplexy, JESS score, sleep onset latency, and the number of SOREMPs on MSLT, and the ADC and FA values for any brain areas in the NA/CA group.

## Discussion

Some previous voxel-based morphometry (VBM) studies indicated a volume reduction in the hypothalamus of patients with narcolepsy [7]. Significant neuronal loss in this area was also reported by MR spectroscopy in patients with narcolepsy-cataplexy [18]. In the present study, however, no microstructural changes were observed in the hypothalamus of patients with narcolepsy. Thannickal et al. reported an 85–95% reduction in the number of hypocretin neurons in the hypothalamus along with gliosis in patients with narcolepsy compared to normal population [19], [20]. Considering that hypocretin neurons are distributed mainly in the hypothalamic area but do not form a nucleus [21], the degenerative loss of hypocretin neurons with gliosis in the hypothalamus of patients with narcolepsy is thought to be undetectable by millimeter-sized voxel-based MRI. This speculation has been supported by a study in which no significant structural changes were observed in the hypothalamus by using both VBM and small-volume correction analysis in patients with hypocretin-deficient narcolepsy [8]. Recently, a neuroimaging study used DTI indicated increased mean diffusivity (MD) without FA changes in the hypothalamus of the patients having narcolepsy with cataplexy, suggesting an increase in extracellular fluid space without further deformation [22]. In contrast, another DTI study showed a decreased FA value in the hypothalamus and brainstem in narcolepsy with cataplexy [23]. Meanwhile, our study did not show any abnormality in diffusion indices (ADC and FA) in the hypothalamus or brainstem. This discrepancy among the study results may be attributable to the differences in the method of calculating the diffusion index (MD or ADC), and/or to differences in the populations tested. In the previous 2 studies, the patients with narcolepsy had been receiving psychostimulants for several years and were older (average age: 56.9 years and 49.5 years, disease duration: 30.6 years and 21.8 years, respectively) compared to participants of the present study. As reported earlier, effects of both of age [24] and medication [25] should be considered when interpreting DTI results.

Several studies have reported an amygdala dysfunction in narcolepsy-cataplexy[26], [27]. In the canine model of narcolepsy, neurodegeneration with gliosis was found in the amygdala[28], and abnormal activity of amygdala neurons was suggested to be related to the occurrence of cataplexy [29]. ADC values represent a tissue water diffusivity and a high ADC value may reflect several conditions due to vasogenic edema associated with increased extracellular space [13]. ADC values are also known to reflect changes in the histopathologic progression; i.e., a low ADC value indicates the presence of cytotoxic edema and cell injury leading to inflamation and cell death, and a high ADC value reflects necrosis, degeneration, and demyelination [30]. Recently, a study on human subjects fond that a high ADC value also represented compensatory gliosis due to vasogenic edema [31], and another study, using an animal model, reported associated of a high ADC value with gliosis following hypoxia-induced ischemia [32]. Similar to previous reports using different techniques [26], [27], our study showed some microstructural abnormalities (high ADC value) in the left amygdala in the NA/CA group, and no changes the NA w/o CA group. The left amygdala has been suggested to play a role in emotional interference resolution by processing arousing verbal stimuli, while the right amygdala processes non-verbal stimuli [33]. Considering these previous suggestions, we think that our result of microstructural abnormalities in the left amygdala might be associated with compensatory hyperactivities in the right amygdala, as reported by Schwartz S et al [27].

A previous VBM study indicated gray matter volume reduction in the inferior frontal gyrus in narcolepsy with cataplexy, and the authors speculated that hypocretin neuron loss might lead to some degree of cortical neuroglial atrophy and cortical volume loss [34]. In contrast, our results showed a higher ADC value for the right inferior frontal gyrus. Although the reasons for such differences remain unclear, differences in treatment and disease duration between the 2 studies should be considered. Regarding this, although the NA/CA participants in our study were younger (29.4+/−4.9 years old) than those in the study by Kaufmann et al. [34] (36.9+/−5.8 years old), we did not find any significant correlations between the neuroimaging findings, disease duration, and age at onset of narcolepsy. Kaufmann et al. [34] also reported that the volume reduction in the gray matter was independent of disease duration. The influence of medication, mainly antidepressants and psychostimulants, taken for years in their study [34], may have affected the neuroimaging results of loss of cortical volume in contrast to our study.

In the present study, the NA/CA and NA w/o CA groups showed morphological differences; the 2 groups differed in the ADC values for the right inferior frontal gyrus and FA values for the right precuneus. Although it is still debatable, Thannickal et al. reported decreased hypocretin fiber density throughout the hypothalamus in narcolepsy with cataplexy and partial (33%) loss of hypocretin cells in the posterior hypothalamus in narcolepsy without cataplexy [12]. These findings suggest that symptomatic differences between narcolepsy with and without cataplexy are related to either the severity and localizations of hypocretin neurons loss or a different pathway. Since hypocretin neuron projections are widely distributed over the CNS, including the cerebral cortex [35], cerebral cortex abnormalities in the NA/CA group observed in our study might be related to hypocretin neuron loss. If that is the case, then the lack of any morphological abnormalities in the cortex in the NA w/o CA group suggests the absence of, or perhaps limited, hypocretin cell loss. Recently, Bayard et al. reported slower performance and more variable results on simple reaction time tasks for patients having narcolepsy with cataplexy compared to those having narcolepsy without cataplexy [36]. Some studies have suggested that the right inferior frontal gyrus plays a predominant role in executive control [37], and the precuneus as well as the dorsolateral prefrontal cortex, is associated with executive control of attention shifts [38]. Thus, the right inferior frontal gyrus and the right precuneus abnormalities, along with a modest change in the executive network area, identified in the present study, may reflect the attention dysfunction in NA/CA. Our findings also favor the notion that cataplexy is related to abnormal interactions between the amygdala, post-central gyrus (Brodmann area 3), which is associated with motor control, and inferior frontal gyrus, which is associated with the production of emotions, since abnormal FA and ADC values in these areas were observed only in the NA/CA group.

Previous studies reported milder severity of EDS, lower REM propensity and less disturbed nocturnal sleep in patients having narcolepsy without cataplexy than those having narcolepsy with cataplexy [10], [11]. Short sleep latencies and multiple SOREMPs on the MSLT, suggesting a narcolepsy-like phenotype, may be observed in several conditions, including shift-work disorder and behaviorally induced insufficient sleep syndrome, as well as in cases of obstructive sleep apnea syndrome, low nocturnal oxygen saturation, and antidepressant drug intake [39], [40]. Altogether, these findings favor that narcolepsy without cataplexy is not a homogeneous entity, unlike narcolepsy with cataplexy which may be also an unstable and reversible condition. Accordingly, we failed to find any neuroimaging differences between the NA w/o CA and NC groups.

This study has several limitations. First, we did not measure CSF-hypocretin-1 levels in the patients. However, we assume that almost all patients with typical sporadic NA/CA were hypocretin deficient, while 80–90% of the patients with a long disease history of NA w/o CA had normal CSF-hypocretin-1 levels [9]. Thus, we hypothesize that brain morphological differences noted between the NA/CA and NA w/o CA groups relate to hypothalamic hypocretin dysfunction. Second, the duration of disease in both patients groups was over 10 years on average, so potential morphological abnormalities observed in this study might involve compensatory changes that had occurred in response to the basic pathology of narcolepsy. Third, similar to previous neuroimaging studies, the present study recruited relatively few patients in the NA/CA and NA w/o CA groups, which may cause a type I error, possibly contributing to some conflicting results between studies.

In summary, our study has demonstrated that brain morphometric abnormalities in the left amygdala, left inferior frontal gyrus, and left postcentral gyrus exist in narcolepsy with cataplexy, but not in narcolepsy without cataplexy. These findings suggest that these 2 types of narcolepsy have different pathological mechanisms. Further studies are needed to investigate the cause of neuronal alterations in the brain areas involved in narcolepsy with cataplexy and to validate the absence of neuroimaging biomarkers in narcolepsy without cataplexy.
